# Prognostic Biomarker SLCO4A1 Is Correlated with Tumor Immune Infiltration in Colon Adenocarcinoma

**DOI:** 10.1155/2023/4926474

**Published:** 2023-04-17

**Authors:** Xiaolong Chen, Gangfeng Yi, Yu Zhou, Weijun Hu, Lingyun Xi, Weilan Han, Fei Wang

**Affiliations:** Department of General Surgery, Xi'an No. 1 Hospital, The First Affiliated Hospital of Northwestern University, Xi'an, Shaanxi Province, China

## Abstract

**Background:**

Solute carrier organic anion transporter family member 4A1 (SLCO4A1), a member of solute carrier organic anion family, is a key gene regulating bile metabolism, organic anion transport, and ABC transport. However, the association of SLCO4A1 with prognosis and tumor immune infiltration in colon adenocarcinoma (COAD) remains indistinct.

**Methods:**

Firstly, we explored the expression level of SLCO4A1 in COAD via GEPIA, Oncomine, and UALCAN databases. Secondly, we used the Kaplan-Meier plotter and PrognoScan databases to investigate the effect of SLCO4A1 on prognosis in COAD patients. In addition, the correlation between SLCO4A1 and tumor immune infiltration was studied by using TIMER and TISIDB databases.

**Results:**

Our results showed that SLCO4A1 was overexpressed in COAD tissues. At the same time, our study showed that high expression of SLCO4A1 was associated with poor overall survival, disease-free survival, and disease-specific survival in COAD patients. The expression level of SLCO4A1 was negatively linked to the infiltrating levels of B cells, CD8+ T cells, and dendritic cells in COAD. Moreover, the expression of SLCO4A1 was significantly correlated with numerous immune markers in COAD.

**Conclusions:**

These results indicated that SLCO4A1 could be associated with the prognosis of COAD patients and the levels of tumor immune infiltration. Our study suggested that SLCO4A1 could be a valuable biomarker for evaluating prognosis and tumor immune infiltration in COAD patients.

## 1. Introduction

In recent decades, colorectal cancer (CRC) has become one of the most common malignancies in the world [1]. Morbidity and mortality of CRC are increasing every year, particularly in developing countries, where the morbidity and mortality of CRC increase by about 20 percent per year [2]. The disease has emerged as one of the major challenges facing global health. Although there are significant advances in cancer diagnosis and treatment, overall survival (OS) in CRC patients remains unsatisfactory. When many CRC patients are diagnosed, their tumors are already in the middle and advanced stage, and the patients often have regional lymph node metastasis or distant organ metastasis. Therefore, there is an urgent need to understand the pathogenesis of CRC and to identify potential biomarkers to assess the prognosis and treatment effect of CRC patients.

Immunotherapy is the most promising treatment for colorectal cancer, especially for the microsatellite instability-high (MSI-H) phenotype [3–5]. MSI-H colorectal cancer has many mutations that produce many new antigens, stimulating tumor immune infiltration and improving immune checkpoint suppression [6, 7]. Programmed death ligand-1 (PD-L1) is an important target of tumor immunotherapy in clinical trials and has a significant therapeutic effect on liver hepatocellular carcinoma (LIHC) and non-small-cell lung cancer (NSCLC) [8, 9]. Numerous studies have confirmed that tumor-infiltrating immune cells (TIICs), especially cytotoxic T lymphocyte (CD8+ T) cells, significantly affect the prognosis of cancer patients and the effect of immunotherapy and chemotherapy [10–12]. For example, CD8+ tumor-infiltrating lymphocyte was found to be positively associated with PD-L1 status in colorectal cancer patients [13]. However, clinical immunotherapy outcomes show that many monoclonal antibodies have poor clinical efficacy in advanced colorectal cancer, although anti-PD-1 and anti-PD-L1 monoclonal antibodies have obvious efficacy in the treatment of metastatic colorectal cancer [14–17]. There is an urgent need to explore new biomarkers to better assess the prognosis of CRC patients and to identify novel immune-based therapeutic targets.

Solute carrier organic anion transporter family member 4A1 (SLCO4A1), also known as organic anion-transporting polypeptide 4A1 (OATP4A1), is an important member of solute carrier organic anion transporter (SLCO) family, responsible for the Na^+^-independent transmembrane transport of many substrates, such as many drugs, thyroid hormone, some toxins [18]. The changes in the uptake of these substrates may lead to variations in the concentration of anticancer drugs in cancer cells, thus playing an important role in the chemical sensitivity of cancer cells and influencing tumor progression [19]. For example, cisplatin activates SLCO4A1 and affects the progression and metastasis of lung cancer NCIH417 cells [20]. In addition, SLCO4A1 has been found to be overexpressed in pancreatic cancer and is expected to be an important biomarker for targeted therapy of pancreatic cancer [21]. At present, the biological role of SLCO4A1, its prognostic value, and the relationship of SLCO4A1 with tumor immune infiltration in COAD are still unclear.

In this study, the expression level and prognostic value of SLCO4A1 in patients with COAD were analyzed using multiple bioinformatic databases, such as Oncomine, UALCAN, PrognoScan, GEPIA, and Kaplan-Meier plotter. Using the interactive online websites STRING and OmicShare tools, the functional enrichment analysis was conducted to explore the potential molecular mechanism of SLCO4A1 in the progress of COAD. In addition, the relationship of SLCO4A1 with tumor immune infiltration in COAD was verified via TIMER and TISIDB databases. Our study examined whether SLCO4A1 could be used as an important biomarker to evaluate the prognosis and the efficacy of immunotherapy in COAD patients.

## 2. Materials and Methods

### 2.1. Bioinformatic Analysis of SLCO4A1 Expression

Oncomine (http://www.oncomine.org/) is currently the world's largest oncogene chip database and integrated data mining platform [22, 23]. We compared the mRNA expression of SLCO4A1 in COAD tissues and matched normal tissues using the Oncomine database. Firstly, we enter SLCO4A1 into the search box to get the expression profile of SLCO4A1 for various cancers. Secondly, tumor vs. normal analysis was used, and the tumor type was selected as COAD. Then, we set *P* < 0.05 and fold change > 1.5. Finally, the statistical values were obtained from the analysis results of related databases. UALCAN (http://ualcan.path.uab.edu/index.html/) is a powerful online database for analyzing cancer-related data, and we assessed the expression of SLCO4A1 in COAD via the UALCAN database [24]. Moreover, we investigated the relationship of different clinical features with the expression level of SLCO4A1, such as race, sex, weight, age, lymph node metastasis, individual cancer stage, histological subtype, and TP53 mutation. In addition, we further explored the relationship between the mutation status of seven important clinically detected proteins (MLH1, PMS2, MSH2, MSH6, BRAF, KRAS, and NRAS) and the expression of SLCO4A1 by TIMER (http://timer.cistrome.org/) [25]. Immunohistochemical staining for the SLCO4A1 protein in COAD tissue was obtained from the Human Protein Atlas (https://www.proteinatlas.org/). The three-dimensional structural model of the SLCO4A1 protein was constructed by using the SWISS-MODEL (https://swissmodel.expasy.org/) [26]. We followed the methods of Huang et al. [27].

### 2.2. Prognostic Survival Analysis

The prognostic value of SLCO4A1 in COAD patients was studied by using GEPIA, PrognoScan, and Kaplan-Meier plotter. GEPIA (http://gepia.cancer-pku.cn/) is a concise, easy-to-use platform for analyzing the relationship between SLCO4A1 and survival in COAD patients [28, 29]. PrognoScan (http://www.prognoscan.org/) is a new online platform that can predict the association of different genes with cancer patients' prognosis [30]. The Kaplan-Meier plotter (https://kmplot.com/analysis/) is an online database to assess the effect of targeted genes on the survival in 21 cancer types and is used to further validate our survival analysis [31]. COAD patients were divided into two groups based on their expression level of the SLCO4A1 gene. Survival analysis was evaluated using the hazard ratios (HRs) and *P* value, with *P* = 0.05 being the cut-off value for significance. We followed the methods of Huang et al. [27].

### 2.3. cBioPortal Database

Using the cBioPortal database (https://www.cbioportal.org/), the genetic alterations of the SLCO4A1 gene were explored [32]. Firstly, we selected three study datasets “DFCI, Cell Reports 2016,” “TCGA, Firehose Legacy,” and “TCGA PanCancer Atlas” and imported SLCO4A1 into “Gene Symbols” box. Secondly, the structural variation data, mutation data, and CNA data were analyzed separately in the “Tumor Types Summary” module. Thirdly, we also showed the SLCO4A1 gene mutation via a schematic diagram. In addition, we selected “comparison/survival” module to evaluate the effect of SLCO4A1 gene mutation on the survival in COAD patients. We followed the methods of Huang et al. [27].

### 2.4. SLCO4A1-Related Gene Enrichment Analysis

STRING (http://string-db.org/) was used for exploring SLCO4A1 protein-protein interactions [33]. The minimum interaction score required was 0.400, and the maximum number of these interactions was 50. The top 50 interacting proteins of SLCO4A1 were regarded as SLCO4A1-binding proteins. Furthermore, we clicked SLCO4A1 on the “Query Search” module to get the top 100 SLCO4A1-related genes via GEPIA. OmicShare tools (http://omicsshare.com/tools) was an efficient online platform which was used for functional enrichment analysis of SLCO4A1-related genes [34].

### 2.5. Analysis of Tumor Immune Infiltration

TIMER (https://cistrome.shinyapps.io/timer/) is a comprehensive online platform for immune infiltration analysis in various tumors [35]. TIMER uses various deconvolutional statistical methods to predict the abundance of infiltrating immune cells in different tumors. In this study, we explored the relationship between the SLCO4A1 expression and tumor-infiltrating immune cell abundance in COAD patients using TIMER. Tumor-infiltrating immune cells (TIICs) are mainly composed of CD8+ T cells, CD4+ T cells, B cells, neutrophils, natural killer cells, dendritic cells, monocytes, macrophages, and T-helper cells. Molecular markers of these immune cells have been used in many previous studies [36–38]. The “Correlations” module may produce scatterplots of Spearman correlation for an interesting pair of genes in a specific cancer type. The expression level of specific genes was represented with log_2_ RSEM.

TISIDB (http://cis.hku.hk/TISIDB/index.php/), an open online platform to explore the interaction of tumors and immune system, was used for analyzing the relationship between SLCO4A1 expression levels and different immune components [39].

## 3. Results

### 3.1. Overexpression of SLCO4A1 in COAD

**Figure 1:**
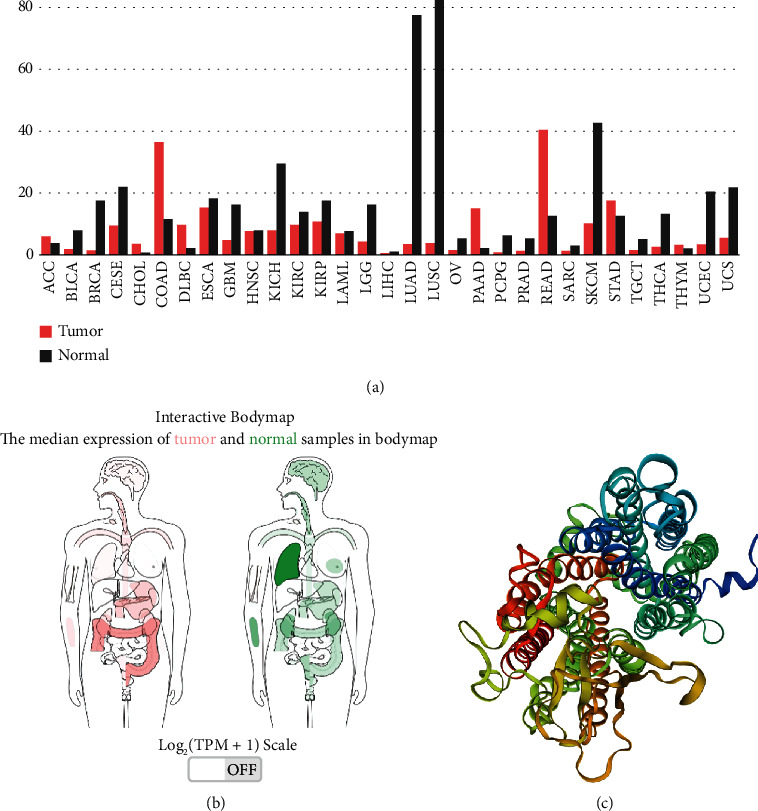
Expression levels of SLCO4A1 in a variety of cancers and its 3D protein model structure. (a) Expression levels of SLCO4A1 in different cancer samples were investigated via the GEPIA database. (b) The interactive bodymap of SLCO4A1 was shown using the GEPIA database. (c) The 3D structure of the SLCO4A1 protein was constructed via the SWISS-MODEL.

The expression level of SLCO4A1 in human tumors was analyzed via the GEPIA database. The results showed that SLCO4A1 was downregulated in BLCA, BRCA, CESC, SLCO4A1, CHOL, ESCA, GBM, HNSC, KIRC, LUAD, LUSC, PCPG, and SARC while SLCO4A1 was significantly upregulated in COAD, READ, PAAD, and STAD (Figure 1(a)). An interactive bodymap of SLCO4A1 is shown in Figure 1(b). In addition, the 3D structural model of the SLCO4A1 protein was constructed by using the SWISS-MODEL (Figure 1(c)). The abbreviation of each tumor is shown in Supplementary Table 1.

**Figure 2:**
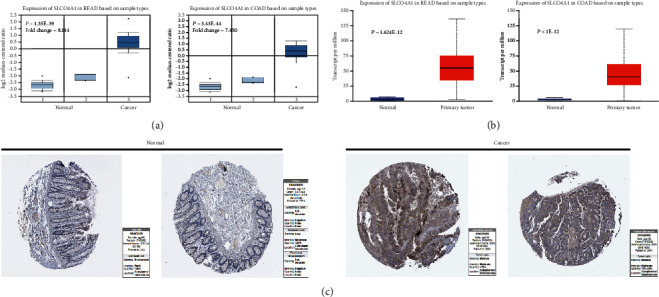
SLCO4A1 expression levels in CRC samples. (a) SLCO4A1 expression levels in COAD and READ samples were explored via the Oncomine database. (b) SLCO4A1 expression levels in COAD and READ were explored using the UALCAN database. (c) Immunohistochemical images of SLCO4A1 protein in COAD and READ tissues from the Human Protein Atlas.

Moreover, we also used Oncomine and UALCAN databases to detect the mRNA expression level of SLCO4A1 in COAD. SLCO4A1 was highly expressed in colorectal cancer (including COAD and READ) compared with the corresponding normal tissues (Figures 2(a) and 2(b)). In addition, the expression level of the SLCO4A1 protein was elevated in COAD tumor tissues obtained from the Human Protein Atlas (Figure 2(c)).

### 3.2. The Relationship between the Expression Level of SLCO4A1 and Clinical Characteristics in COAD

In this study, the UALCAN database was used to explore the relationship between the expression level of SLCO4A1 and clinical characteristics in COAD. Different race, gender, weights, age, and nodal metastasis status were not linked to SLCO4A1 mRNA expression in patients with COAD. However, in terms of individual cancer stages, stage 1 group had a lower expression level than the stage 3 or 4 group. Mucinous adenocarcinoma presented with higher SLCO4A1 expression than adenocarcinoma in patients with COAD. Furthermore, SLCO4A1 had a higher expression level in those COAD tissues carrying a TP53 mutation. The relationships between the expression level of SLCO4A1 and clinical characteristics in COAD are shown in Table 1. In addition, the SLCO4A1 expression level was significantly related to the mutation status of MSH2 (*P* = 0.01) and BRAF (*P* = 0.036), but was not linked to the mutation status of MSH6 (*P* = 0.098), PMS2 (*P* = 0.81), MLH1 (*P* = 0.9), KRAS (*P* = 0.39), or NRAS (*P* = 0.67) (Supplementary Figure 1A–1G).

### 3.3. Correlation between the Expression of SLCO4A1 and Prognosis in COAD

**Figure 3:**
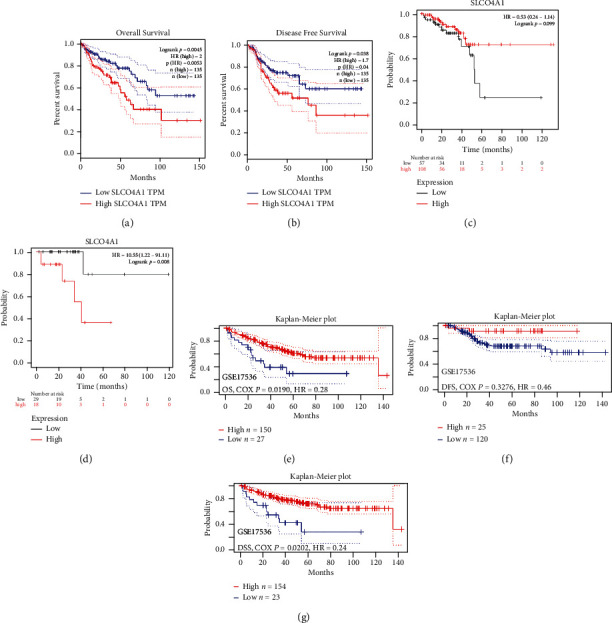
The relationship between SLCO4A1 expression levels and COAD patients' prognosis. (a) OS and (b) DFS of COAD patients based on SLCO4A1 expression levels via GEPIA. (c) OS and (d) DFS of COAD patients based on SLCO4A1 expression levels via the Kaplan-Meier plotter. (e) OS, (f) DFS, and (g) DDS of COAD patients based on SLCO4A1 expression levels via the PrognoScan database. OS: overall survival; DFS: disease-free survival; DSS: disease-specific survival.

The prognostic role of SLCO4A1 in COAD patients was explored via several databases. Overexpression of SLCO4A1 was associated with shorter OS (*P* = 135, HR = 2, *P* = 0.0045) using GEPIA (Figure 3(a)). Moreover, there were significant differences in DFS (*n* = 135, HR = 1.7, *P* = 0.038) (Figure 3(b)). Although SLCO4A1 was not associated with prolonged OS (*n* = 165, HR = 0.53, *P* = 0.099), it was significantly correlated with DFS (*n* = 47, HR = 10.55, *P* = 0.008) via the Kaplan-Meier plotter (Figures 3(c) and 3(d)).

The correlation between the expression of SLCO4A1 and prognosis in COAD patients was further explored using the PrognoScan database. The results showed that overexpression of SLCO4A1 was associated with shorter OS (*n* = 177, HR = 0.0, cox-*P* = 0.019) and DSS (*n* = 177, HR = 0.24, cox-*P* = 0.0202) in COAD patients (Figures 3(e)–3(g)) but no significant difference in DFS (*n* = 145, HR = 0.46, cox-*P* = 0.3276) (Figure 3(f)).

In short, based on these databases, this study explored the correlation between the expression of SLCO4A1 and prognosis in COAD patients and found that SLCO4A1 was a valuable biomarker for evaluating prognosis in patients with COAD.

### 3.4. Genetic Alterations of SLCO4A1 in COAD

**Figure 4:**
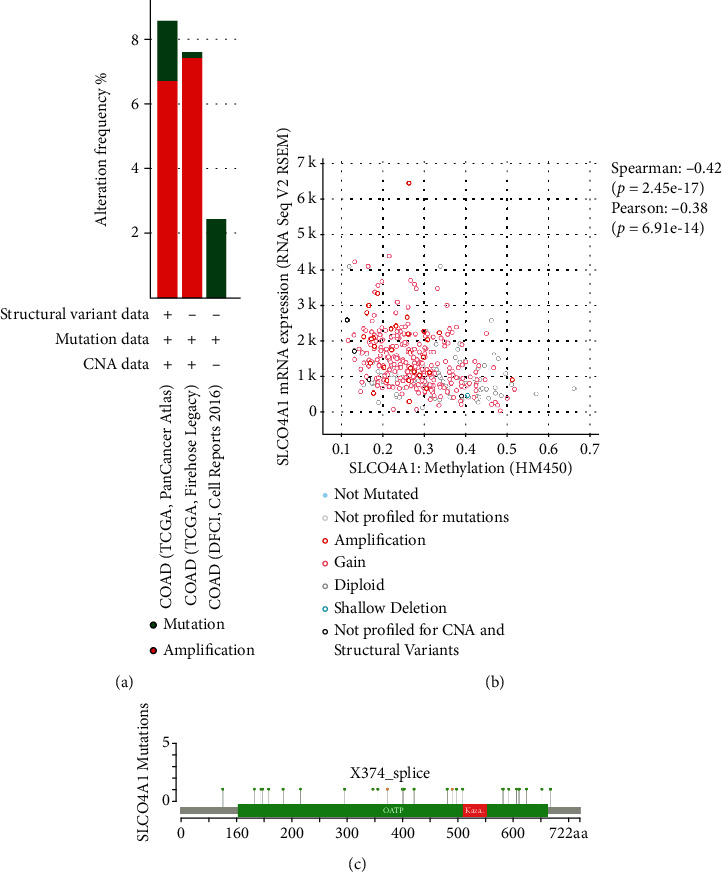
Genetic alterations of SLCO4A1 in COAD. (a) Alteration frequency of SLCO4A1 gene in three COAD studies. (b) Methylation levels of SLCO4A1 based on 372 samples with data in both axes. (c) Mutation diagram showing mutation sites, mutation types, and the number of cases; these results are colored according to the corresponding mutation type.

Numerous studies have shown that genetic variation plays a pivotal role in the pathogenesis and development of various tumors. Our study investigated the genetic alterations of SLCO4A1 via the cBioPortal database. The results showed that somatic mutation of SLCO4A1 was present in about 7.6% of COAD samples (Figure 4(a)). In COAD, copy number variation (CNV) was the main mutation type of SLCO4A1 genetic alterations. The mutation types and the proportions of these mutations are shown in Figure 4(b). Information such as mutation site, mutation type, and number of cases was displayed on the mutation diagram and colored according to the corresponding mutation type (Figure 4(c)). In addition, we also explored the relationship between SLCO4A1 genetic alterations and COAD patients' survival. However, our study showed that SLCO4A1 gene alterations were not associated with OS (*P* = 0.664), PFS (*P* = 0.528), DFS (*P* = 0.882), or DSS (*P* = 0.946) in COAD patients (Supplementary Figure 2A–2D).

### 3.5. GO and KEGG Enrichment Analyses of SLCO4A1

**Figure 5:**
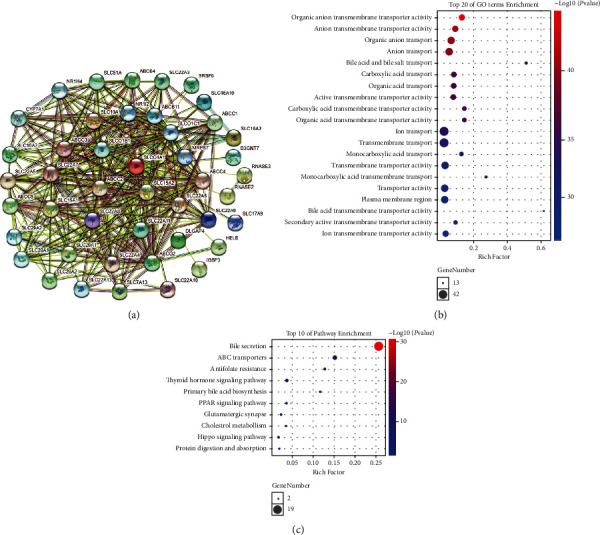
The protein-protein interaction network of SLCO4A1 and functional enrichment analysis. (a) The protein-protein interaction network based on SLCO4A1-binding proteins using the STRING tool. (b) GO enrichment analysis (top 20 terms) by genes binding to SLCO4A1. (c) KEGG enrichment analysis (top 10 terms) by genes binding to SLCO4A1.

In this study, the binding proteins of SLCO4A1 and the genes related to SLCO4A1 expression were identified using the STRING and GEPIA databases. The top 50 binding proteins of SLCO4A1 and the top 100 genes related to the expression of SLCO4A1 are summarized in Supplementary Table 2, and SLCO4A1-binding protein interacting network is shown in Figure 5(a). Furthermore, we also used the SLCO4A1-binding proteins to explore GO enrichment analysis and KEGG pathway analysis. GO enrichment analysis showed that these genes were obviously enriched in organic anion transmembrane transporter activity, anion transmembrane transporter activity, organic anion transport, anion transport, bile acid and bile salt transport, carboxylic acid transport, organic acid transport, active transmembrane transporter activity, carboxylic acid transmembrane transporter activity, organic acid transmembrane transporter activity, ion transport, transmembrane transport, monocarboxylic acid transport, transmembrane transporter activity, monocarboxylic acid transmembrane transport, transporter activity, plasma membrane region, bile acid transmembrane transporter activity, secondary active transmembrane transporter activity, and ion transmembrane transporter activity (Figure 5(b)). In addition, KEGG pathway analysis found that SLCO4A1-interacting proteins were enriched in bile secretion, ABC transporters, antifolate resistance, thyroid hormone signaling pathway, primary bile acid biosynthesis, PPAR signaling pathway, glutamatergic synapse, cholesterol metabolism, hippo signaling pathway, and protein digestion and absorption (Figure 5(c)).

### 3.6. Correlation between SLCO4A1 and Tumor Immune Infiltration in COAD

**Figure 6:**
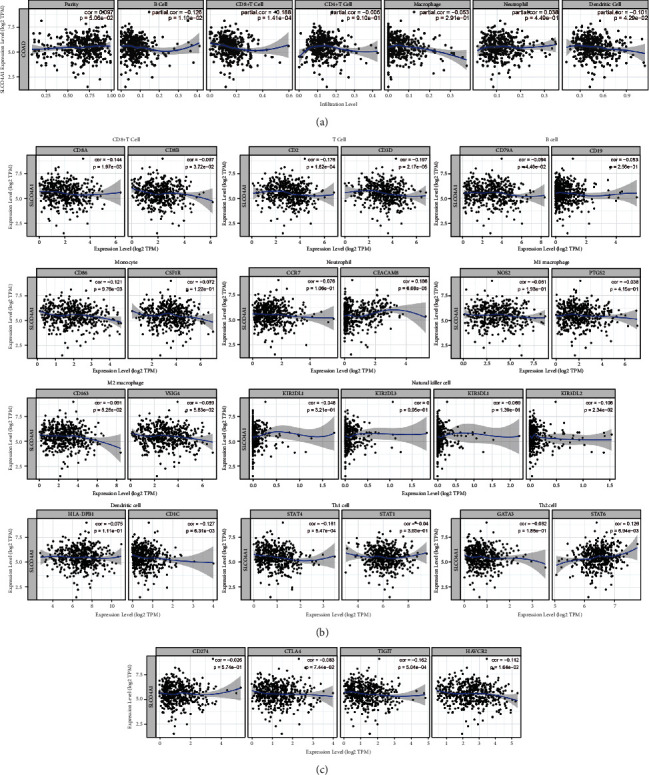
Correlation of the SLCO4A1 expression level with immune infiltration in COAD via the TIMER database. (a) Correlation of the SLCO4A1 expression with different tumor-infiltrating immune cells. (b) Correlation between SLCO4A1 expression and immune cell-specific markers. (c) Correlation between SLCO4A1 expression and four important immune markers (CD274, CTLA4, TIGIT, and HAVCR2).

The correlation of the expression level of SLCO4A1 with tumor immune infiltration in COAD was investigated using the TIMER database. Our study found that the expression level of SLCO4A1 gene was closely related to B lymphocytes (cor = −0.126, *P* = 1.10*e* − 02), CD8+ T lymphocytes (cor = −0.188, *P* = 1.41*e* − 04), and dendritic cells (cor = −0.101, *P* = 4.29*e* − 2) (Figure 6(a)). However, the expression of SLCO4A1 was not associated with tumor purity (cor = 0.097, *P* = 5.06*e* − 2), CD4+ T cells (cor = −0.006, *P* = 9.10*e* − 1), macrophages (cor = −0.053, *P* = 2.92*e* − 1), and neutrophils (cor = 0.038, *P* = 4.49*e* − 1) (Figure 6(a)). So we speculated that these three immune cells (B lymphocytes, CD8+ T lymphocytes, and dendritic cells) were more likely to be responsible for the prognosis and survival difference between patients with different expression of SLCO4A1.

Using the TIMER database, we deeply explored the relationship between SLCO4A1 and immune specific markers. Our study showed that SLCO4A1 was negatively correlated with a large number of immune specific markers, such as CD8A, CD8B, CD3D, CD3E, CD2, CD79A, CD86, KIR3DL2, CD1C, STAT4, and STAT6, and was positively correlated with CEACAM8 (Figure 6(b)). The more detailed results from the database are shown in Table 2. It was suggested that SLCO4A1 could play an important role in regulating immune cell infiltration in COAD. In addition, we further investigated the association of SLCO4A1 with other four important immune markers CD274 (also known as PD-L1), CTLA4, TIGIT, and HAVCR2. There was significant correlation between SLCO4A1 and two immune markers (TIGIT and HAVCR2) but no significant correlation between SLCO4A1 and two other immune markers (CD274 and CTLA4) in COAD (Figure 6(c)).

In this study, the TISIDB database was used to investigate the relationship between the expression of SLCO4A1 in COAD and three immune components (lymphocytes, immunomodulators, and chemokines). Firstly, the relationship between SLCO4A1 expression level and the abundance of tumor infiltrating lymphocytes was explored to identify which types of TIICs could be regulated by SLCO4A1 gene. The results showed that SLCO4A1 expression level was negatively correlated with Tem_CD8 cells (rho = −0.209, *P* = 6.36*e* − 06), Tfh cells (rho = −0.194, *P* = 2.88*e* − 05), Treg cells (rho = −0.164, *P* = 0.00041), Th1 cells (rho = −0.19, *P* = 4.49*e* − 05), Act_CD4 cells (rho = −0.217, *P* = 2.99*e* − 06), and macrophages (rho = −0.266, *P* = 7.72*e* − 09) (Supplementary Figure 3A). Secondly, we identified the correlation between SLCO4A1 expression and immunomodulators (Supplementary Figure 3B–3D). Finally, we investigated the relationships of SLCO4A1 expression with chemokines and receptors. The correlation between SLCO4A1 and chemokines is shown in Supplementary Figure 3E, and the correlation between SLCO4A1 and receptors is shown in Supplementary Figure 3F. These results strongly suggested that SLCO4A1 could regulate a variety of immune components via multiple pathways and then influence tumor immune infiltration in COAD.

## 4. Discussion

COAD is a common and important pathological type in CRC. In the current clinical practice, radical surgery is the only possible cure treatment for most CRC patients. However, surgical operations are sometimes limited or even unable to carry out due to tumor location, depth of tumor invasion, or tumor metastasis. In recent years, new technologies, such as molecular targeted therapy and immunotherapy, have become one of the important means of treating cancer, and the therapeutic effect on some patients with advanced cancer has been significantly improved [40]. As an emerging treatment modality, immunotherapy has become a promising treatment method [41]. However, immunotherapy has only a good response to a small number of CRCs showing microsatellite instability (MSI), but most CRCs belong to the microsatellite stable type (MSS). Compared with MSS tumors, immunostimulatory factors, such as CD28, IL-15, CCL3, and CXCL16, have higher expression in MSI tumors [42]. Activated tumor-infiltrating immune cells could increase the expression level of HLA and checkpoint-related proteins in MSI tumors and then effectively inhibit the immune escape of tumor cells. Therefore, in the future, how to activate immune cells in tumors is an urgent problem to be solved [43, 44].

In recent years, a large number of studies have focused on identifying key immune-related genes in many types of cancers, screening out high-risk populations, and testing the effectiveness of immune-targeted drugs [45–47]. In our study, we found that SLCO4A1 played an important role in the prognosis and tumor immune infiltration in COAD.

Firstly, we studied the expression level of SLCO4A1 in COAD and the relationship of SLCO4A1 abnormal expression with clinical characteristics in patients with COAD. SLCO4A1 was overexpressed in COAD and READ, compared with normal tissues (Figures 1 and 2). Moreover, the expression of SLCO4A1 was associated with individual cancer stages, histological subtype, and TP53 mutation status in patients with COAD (Table 1). In addition, SLCO4A1 overexpression was associated with a shorter OS or DFS (Figure 3). Interestingly, our results are similar to those of some studies, but the role of SLCO4A1 in the occurrence and development of COAD still needs to be further researched [48, 49].

The results of our study showed that the expression level of SLCO4A1 increased in COAD, which affected the prognosis of COAD patients, indicating that SLCO4A1 could play an important role in the pathogenesis of COAD. SLCO4A1 was a valuable research topic not only in genetic alterations (Figure 4) but also in the role of the occurrence and development of COAD. Previous studies have shown that abnormalities of the SLCO4A1 gene have multifaceted effects for many tumors. Overexpression of the SLCO4A1 gene in prostate cancer and thyroid cancer indicated a poor prognosis [48, 50]. Buxhofer-Ausch et al. have shown that SLCO4A1 may affect the accumulation of anticancer drugs in specific cancer cells [19].

Therefore, we hypothesized that SLCO4A1 could be an oncogene in COAD. Then, GO enrichment analysis showed that SLCO4A1-interacting genes were mainly enriched in organic anion transmembrane transporter activity, anion transmembrane transporter activity, bile acid and bile salt transport, carboxylic acid transport, active transmembrane transporter activity, ion transport, monocarboxylic acid transport, plasma membrane region, and secondary active transmembrane transporter activity (Figure 5(b)). The KEGG pathway analysis showed that SLCO4A1 and its coexpressed genes were mainly enriched in bile secretion, ABC transporters, antifolate resistance, thyroid hormone signaling pathway, primary bile acid biosynthesis, PPAR signaling pathway, glutamatergic synapse, cholesterol metabolism, hippo signaling pathway, and protein digestion and absorption (Figure 5(c)). The organic anion transmembrane transport mediates the uptake of many important drugs and hormones, thus affecting the drug distribution and intracellular drug concentration [51]. Because many anticancer drugs are the substrates of SLCOs, the abnormal expression of these transporters in cancer cells will affect the intracellular concentration of anticancer drugs and then affect the efficacy of these drugs. In addition, these influx transporters, which can act together with efflux transporters and drug metabolic enzymes, may play a key role in chemoresistance.

Another major result of our study was that the expression of SLCO4A1 was associated with multiple tumor-infiltrating immune cells and abundant immune molecules (Figure 6). These results strongly suggested that SLCO4A1 could be involved in tumor immune infiltration in COAD. An increased density of CD8+ T cell in tumor tissue has been found to be associated with a reduced risk of tumor recurrence [19]. Tumor-specific CD8+ T cells and CD4+ T cells are required for the effective clearance of tumor cells. We inferred that the suppression of CD8+ T cells could downregulate some important signals on immune cells and then reduce the aggregation of other immune cells, such as CD4+ T cells and dendritic cells, which could explain the simultaneous inhibition phenomenon of several tumor-infiltrating immune cells in our study. Furthermore, the correlation analysis obtained from the TISIDB database revealed the relationship between SLCO4A1 expression levels and lymphocyte, immunomodulators, and chemokines in COAD (Supplementary Figure 3). Our study clearly showed that SLCO4A1 was closely linked to tumor immune infiltration in COAD and might be a new molecular target, which was worth further exploration.

## 5. Conclusions

In conclusion, our study showed that SLCO4A1 was overexpressed in COAD tissues, and we have identified the relationship between SLCO4A1 overexpression and poor prognosis by using several authoritative databases. Moreover, our study also explored the correlation between the expression of SLCO4A1 and tumor immune infiltration in COAD. At the same time, we also studied the association of SLCO4A1 expression with specific markers of diverse immune cells. These results in our study showed that the expression level of SLCO4A1 was significantly related to the abundance of various lymphocytes, immunomodulators, and chemokines in COAD. Therefore, we can better predict the prognosis of COAD and evaluate the status of tumor immune infiltration by testing the expression level of SLCO4A1 in COAD.

There are still some shortcomings in the study. Although the study was based on multiple publicly authoritative databases, the same data in individual research aspects were still limited. In addition, we need to further explore the role of SLCO4A1 in regulating tumor immune infiltration in COAD. However, our current study strongly suggests that SLCO4A1 could be a novel prognostic biomarker and an important immune-related factor for evaluating the immunotherapy in COAD patients.

## Figures and Tables

**Table 1:** SLCO4A1 expression based on different clinical indicators.

Clinical indicators	Number of patients	Comparison	*P* value
Individual cancer stages	45/110/80/39 (stage 1/stage 2/stage 3/stage 4)	Stage 1 vs. stage 2	2.921600*E* − 01
		Stage 1 vs. stage 3	1.616230*E* − 02
		Stage 1 vs. stage 4	2.207800*E* − 02
		Stage 2 vs. stage 3	9.105500*E* − 02
		Stage 2 vs. stage 4	1.213800*E* − 01

Patient's race	193/55/11 (Caucasian/African-American/Asian)	Caucasian vs. African-American	7.265600*E* − 01
		Caucasian vs. Asian	8.521000*E* − 01
		African-American vs. Asian	9.166000*E* − 01

Patient's gender	156/127 (male/female)	Male vs. female	7.154000*E* − 01

Patient's weight	70/74/56 (normal/extreme_weight/obese)	Normal_weight vs. extreme_weight	8.504800*E* − 01
		Normal_weight vs. obese	8.111000*E* − 01
		Extreme_weight vs. obese	6.531800*E* − 01

Patient's age	12/90/149 (21-40 Yrs/41-60 Yrs/61-80 Yrs)	Age (21-40 Yrs) vs. age (41-60 Yrs)	1.452190*E* − 01
		Age (21-40 Yrs) vs. age (61-80 Yrs)	6.386100*E* − 02

Histological subtype	243/37 (adenocarcinoma/mucinous adenocarcinoma)	Adenocarcinoma vs. mucinous adenocarcinoma	6.228500*E* − 05

Nodal metastasis status	166/70/47 (N0/N1/N2)	N0 vs. N1	1.299810*E* − 01
		N0 vs. N2	6.704200*E* − 02
		N1 vs. N2	2.874600*E* − 01

TP53 mutation status	160/122 (mutant/nonmutant)	Mutant vs. nonmutant	1.982530*E* − 05

**Table 2:** Correlation analysis between SLCO4A1 and related genes and markers of immune cells in TIMER.

Description	Gene markers	cor	*P*
CD8+ T cell	CD8A	-0.144	1.97*E* − 03
	CD8B	-0.097	3.72*E* − 02

T cell	CD3D	-0.197	2.17*E* − 05
	CD3E	-0.150	1.34*E* − 03
	CD2	-0.176	1.62*E* − 04

B cell	CD19	-0.053	2.58*E* − 01
	CD79A	-0.094	4.49*E* − 02

Monocyte	CD86	-0.121	9.76*E* − 03
	CSF1R	-0.072	1.22*E* − 01

TAM	CCL2	-0.072	1.26*E* − 01
	CD68	-0.028	5.46*E* − 01
	IL10	-0.014	1.44*E* − 02

M1 macrophage	INOS (NOS2)	-0.061	1.93*E* − 01
	IRF5	0.115	1.38*E* − 02
	COX2 (PTGS2)	-0.038	4.15*E* − 01

M2 macrophage	CD163	-0.091	5.26*E* − 02
	VSIG4	-0.089	5.83*E* − 02
	MS4A4A	-0.133	4.51*E* − 03

Neutrophils	CD66b (CEACAM8)	0.186	6.08*E* − 05
	CD11b (ITGAM)	-0.032	4.96*E* − 01
	CCR7	-0.076	1.06*E* − 01

Natural killer cell	KIR2DL1	-0.046	3.21*E* − 01
	KIR2DL3	0.000	9.95*E* − 01
	KIR3DL1	-0.069	1.39*E* − 01
	KIR3DL2	-0.106	2.34*E* − 02

Dendritic cell	HLA-DPB1	-0.075	1.11*E* − 01
	HLA-DRA	-0.130	5.52*E* − 03
	BDCA-1 (CD1C)	-0.127	6.31*E* − 03

Th1	STAT4	-0.161	5.47*E* − 04
	STAT1	-0.040	3.39*E* − 01
	TNF-*α* (TNF)	-0.015	7.53*E* − 01

Th2	GATA3	-0.062	1.85*E* − 01
	STAT6	0.126	6.94*E* − 03
	IL13	-0.210	6.62*E* − 01

Tfh	BCL6	0.087	6.17*E* − 02
	IL21	-0.050	2.87*E* − 01

Th17 cell	STAT3	-0.004	9.36*E* − 01
	IL17A	0.098	3.52*E* − 02

Treg cell	FOXP3	-0.100	3.20*E* − 02
	CCR8	-0.145	8.26*E* − 04

## Data Availability

All data generated or analyzed during this study are included in this article (and its supplementary information files).

## Supplementary Materials

Supplementary 1 Table S1: the abbreviations for 31 cancers. Table S2: top 100 expression-correlated genes and 50 SLC04A1-binding proteins.

Supplementary 2 Supplementary Figure 1: relationship between SLCO4A1 expression and clinical molecular indicators in patients with COAD. Different SLCO4A1 expression levels in COAD based on (A) PMS2, (B) MSH2, (C) MSH6, (D) MLH1, (E) BRAF, (F) KRAS, and (G) NRAS, respectively. Supplementary Figure 2: analysis of SLCO4A1 genetic alterations via the cBioPortal database. Relationship of SLCO4A1 with genetic alterations (A) OS, (B) PFS, (C) DFS, and (D) DSS for patients with COAD. OATP: organic anion-transporting polypeptide; OS: overall survival; PFS: progression-free survival; DFS: disease-free survival; DSS: disease-specific survival. Supplementary Figure 3: relationship between SLCO4A1 expression and immune components in patients with COAD, including lymphocytes, immunomodulators, chemokines, and receptors. (A) Relationship between the SLCO4A1 expression level and lymphocytes. (B–D) Relationship between the SLCO4A1 expression level and immunomodulators. (E) Correlation between SLCO4A1 expression level and chemokines. (F) Correlation between SLCO4A1 expression level and receptors.
