# Supplementary material for: Prognostic Biomarker SLCO4A1 Is Correlated with Tumor Immune Infiltration in Colon Adenocarcinoma
Source: Mediators Inflamm. 2023 Apr 17;2023:4926474. doi: 10.1155/2023/4926474 (PMC10137198; doi:10.1155/2023/4926474)
Supplement: Supplementary 1 — Table S1: the abbreviations for 31 cancers. Table S2: top 100 expression-correlated genes and 50 SLC04A1-binding proteins. [file 4926474.f1.docx]

**Supplementary Tables**

| **Table S1.** The abbreviations for 31 cancers | |
| --- | --- |
| Abbreviations | Full name |
| ACC | Adrenocortical carcinoma |
| BLCA | Bladder urothelial carcinoma |
| BRCA | Breast invasive carcinoma |
| CESC | Cervical squamous cell carcinoma |
| CHOL | Cholangiocarcinoma |
| COAD | Colon adenocarcinoma |
| DLBC | Lymphoid Neoplasm Diffuse Large B-cell Lymphoma |
| ESCA | Esophageal carcinoma |
| GBM | Glioblastoma multiforme tumor |
| HNSC | Head and neck squamous cell carcinoma |
| KICH | Kidney chromophobe |
| KIRC | Kidney renal clear cell carcinoma |
| KIRP | Kidney renal papillary cell carcinoma |
| LAML | Acute Myeloid Leukemia |
| LGG | Brain Lower Grade Glioma |
| LIHC | Liver hepatocellular carcinoma |
| LUAD | Lung adenocarcinoma |
| LUSC | Lung squamous cell carcinoma |
| OV | Ovarian serous cystadenocarcinoma |
| PAAD | Pancreatic adenocarcinoma |
| PCPG | Pheochromocytoma and paraganglioma |
| PRAD | Prostate adenocarcinoma |
| READ | Rectal adenocarcinoma |
| SARC | Sarcoma tumor |
| SKCM | Skin cutaneous melanoma |
| STAD | Stomach adenocarcinoma |
| TGCT | Testicular Germ Cell Tumors |
| THCA | Thyroid carcinoma |
| THYM | Thymoma |
| UCEC | Uterine corpus endometrial carcinoma |
| UCS | Uterine Carcinosarcoma |

| **Table S2.** Top 100 expression-correlated genes and 50 SLC04A1-bingding proteins | |
| --- | --- |
| Descriptions | Genes |
| Top 100 expression-correlated genes | SLCO4A1-AS1, MRGBP, OGFR, RP4-655J12.5, GMEB2, TGIF2, SAMD10, ARFRP1, ADRM1, DNAJC5, BCL2L1, ZGPAT, DHX35, TLDC2, OSBPL2, DYNLRB1, ATP11A, DNTTIP1, NPRL3, RPN2, RP11-19N8.7, RP11-586K12.4, RP11-80H8.3, TUBB4BP4, GJA8, AC005514.2, RP11-831A10.2, RP11-806N19.2, KLF18, AIG1P1, BNIP3P18, RP11-359P18.7, HM13, RTFDC1, LINC01216, TAF4, DLGAP4-AS1, ARFGAP1, RAB22A, NDRG3, SS18L1, RALY, NELFCD, STAU1, ACTR5, UBE2V1, CHAMP1, YTHDF1, TTI1, UCKL1, PPDPF, TMC7, NCOA6, DIDO1, TMEM189, FGFR4, AC068538.4, PRPF6, RP4-583P15.15, RP11-93B14.4, SLC35C2, HELZ2, DLGAP4, TCFL5, MTG2, TRPC4AP, MAFG, PSMA7, AAR2, EIF2S2, HSPH1, CBX4, GID8, RP11-314N2.2, SLC1A5, CABLES2, MRPS31P5, DCUN1D2, CYB5B, C20orf24, LSM14B, TPD52L2, SPATA2, TOP1, ATXN2L, NPAS2, TM9SF4, ASXL1, LINC00657, MAPRE1, CUL4A, PPP1R3D, RAE1, AXIN1, EIF6, SLC9A8, RPRD1B, PDPK1, RN7SL495P, RPL5P15 |
| 50 SLCO4A1-bingding proteins | ABCA8, ABCB11, ABCB4, ABCC2, ABCC3, ABCC4, ABCG2, ALB, ATP8B1, CYP7A1, CYP8B1, DIO2, DLG2, DLG3, DLG4, DLGAP4, FABP6, MRPS7, NR0B2, NR1H4, PDZK1, SHANK1, SHANK2, SLC3A1, SLC7A13, SLC10A1, THRA, SLC10A2, SLC10A7, SLC16A10, SLC16A2, SLC16A9, SLC17A1,SLC17A3, SLC22A11, SLC22A7, SLC22A8, SLC22A9, SLC2A9, SLC47A1, SLC47A2, SLC51A, SLC51B, SLCO1A2, SLCO1B1, SLCO1B3, SCLO2B1, SLCO4C1, SLCO1B3-SLCO1B7, SLCO1C1, |
