# Supplementary material for: Prognostic Biomarker SLCO4A1 Is Correlated with Tumor Immune Infiltration in Colon Adenocarcinoma
Source: Mediators Inflamm. 2023 Apr 17;2023:4926474. doi: 10.1155/2023/4926474 (PMC10137198; doi:10.1155/2023/4926474)
Supplement: Supplementary 2 — Supplementary Figure 1: relationship between SLCO4A1 expression and clinical molecular indicators in patients with COAD. Different SLCO4A1 expression levels in COAD based on (A) PMS2, (B) MSH2, (C) MSH6, (D) MLH1, (E) BRAF, (F) KRAS, and (G) NRAS, respectively. Supplementary Figure 2: analysis of SLCO4A1 genetic alterations via the cBioPortal database. Relationship of SLCO4A1 with genetic alterations (A) OS, (B) PFS, (C) DFS, and (D) DSS for patients with COAD. OATP: organic anion-transporting polypeptide; OS: overall survival; PFS: progression-free survival; DFS: disease-free survival; DSS: disease-specific survival. Supplementary Figure 3: relationship between SLCO4A1 expression and immune components in patients with COAD, including lymphocytes, immunomodulators, chemokines, and receptors. (A) Relationship between the SLCO4A1 expression level and lymphocytes. (B–D) Relationship between the SLCO4A1 expression level and immunomodulators. (E) Correlation between SLCO4A1 expression level and chemokines. (F) Correlation between SLCO4A1 expression level and receptors. [file 4926474.f2.docx]

**Supplementary Figures**

**
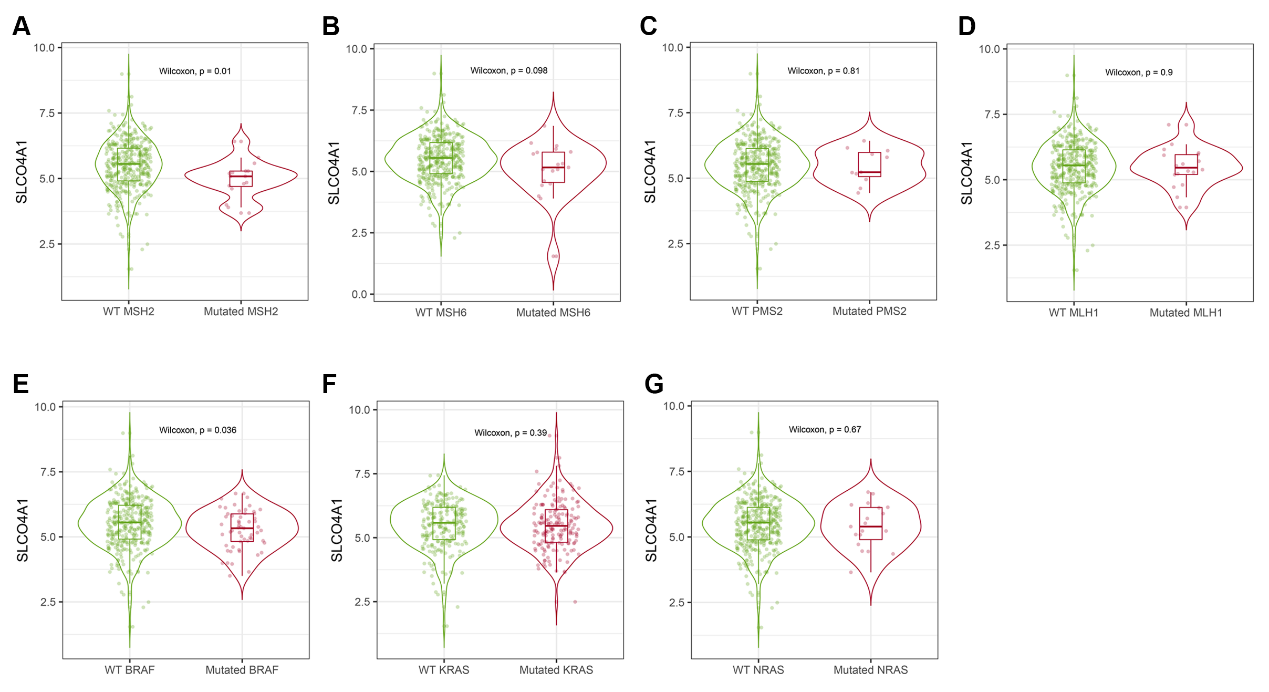
**

**Supplementary Figure 1.** Relationship between SLCO4A1 expression and clinical molecular indicators in patients with COAD. Different SLCO4A1 expression levels in COAD based on (A) PMS2, (B) MSH2, (C) MSH6, (D) MLH1, (E) BRAF, (F) KRAS, and (G) NRAS, respectively.

**
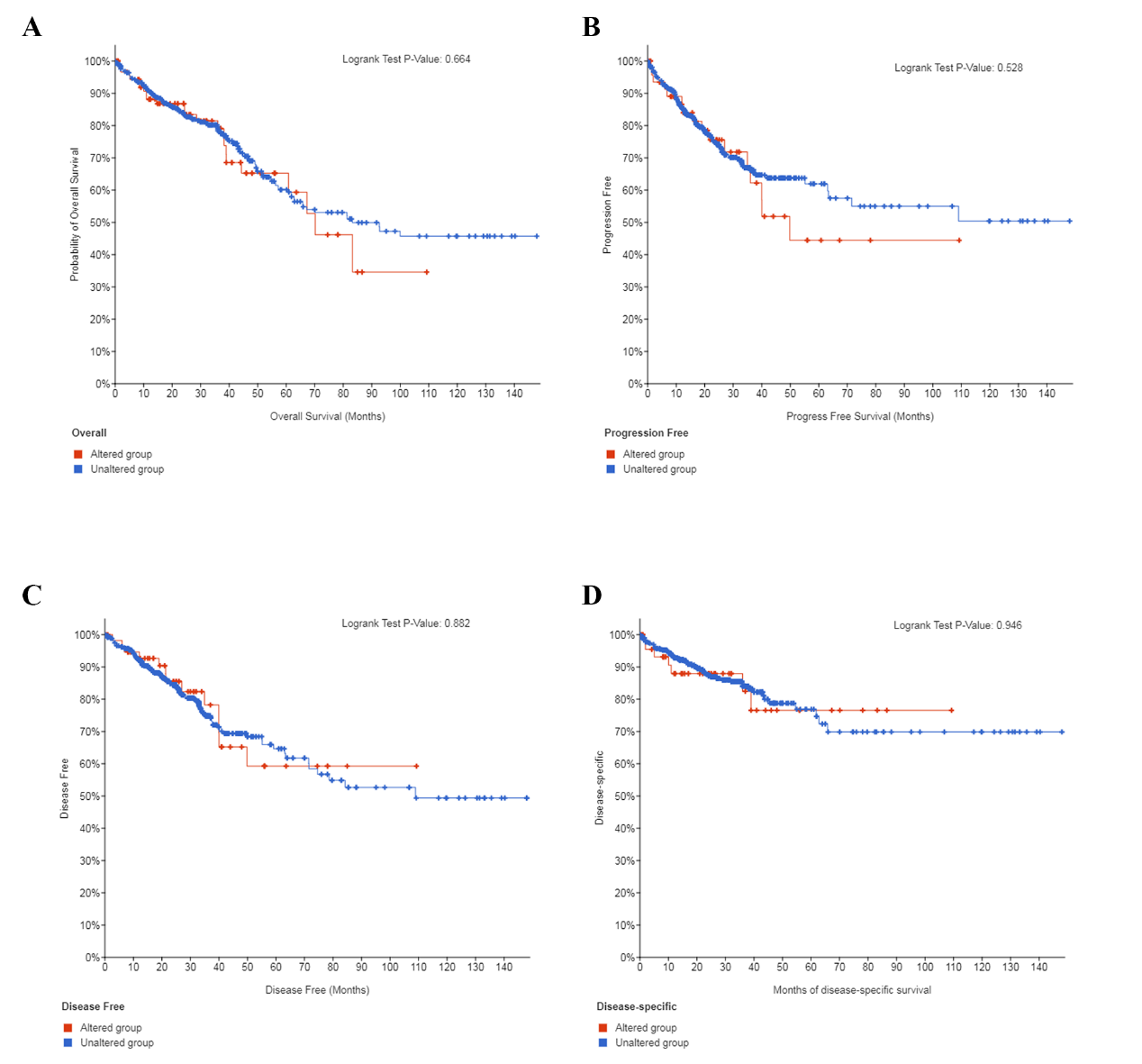
**

**Supplementary Figure 2.** Analysis of SLCO4A1 genetic alterations via the cBioPortal database. Relationship of SLCO4A1 with genetic alterations(A) OS, (B) PFS, (C) DFS, and (D) DSS for patients with COAD. OATP, organic anion transporting polypeptide; OS, overall survival; PFS, progression-free survival; DFS, disease-free survival; DSS, disease-specific survival.

**
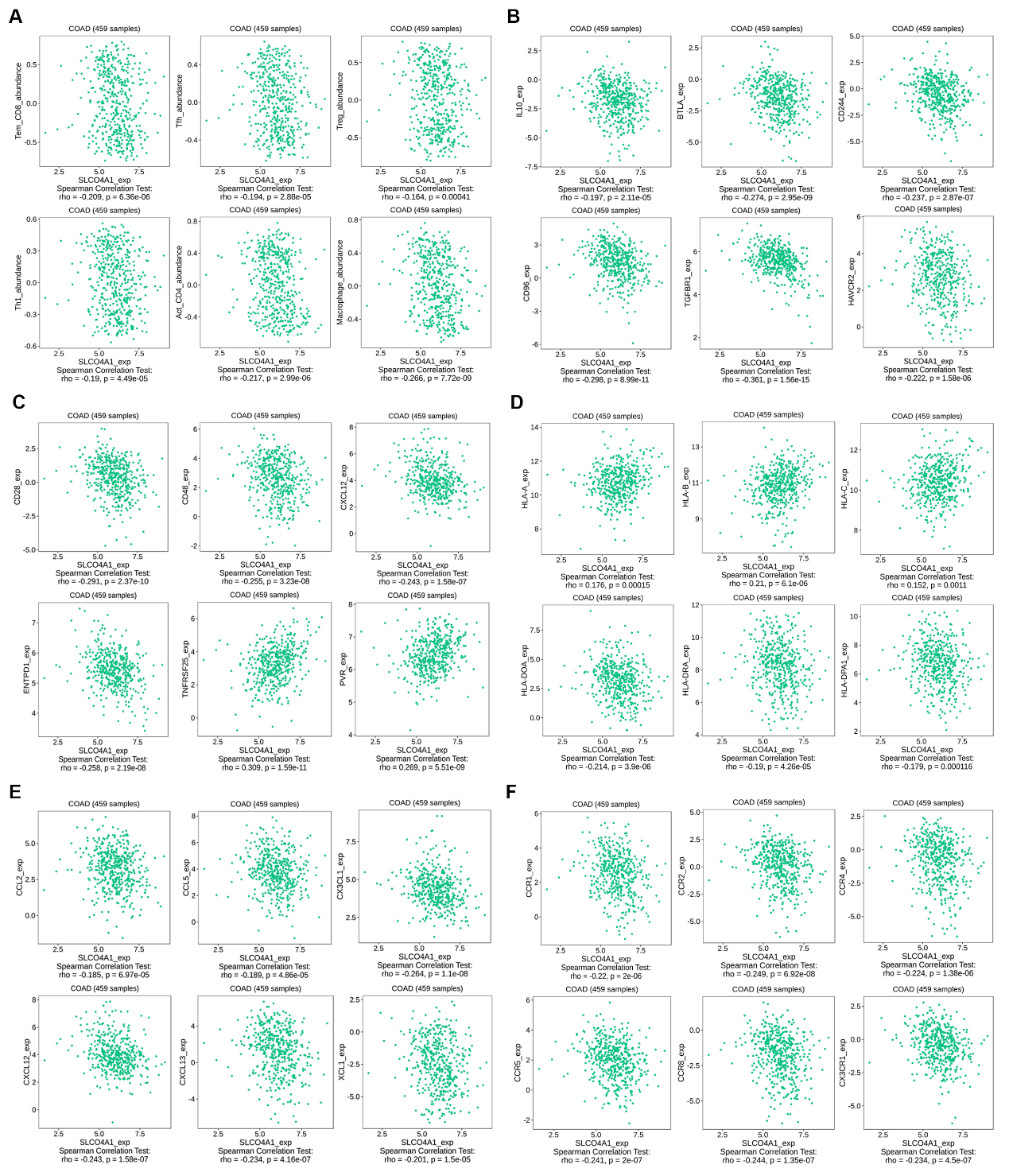
**

**Supplementary Figure 3.** Relationship between SLCO4A1 expression and immune components in patients with COAD, including lymphocytes, immunomodulators, chemokines and receptors. (A) Relationship between the SLCO4A1 expression level and lymphocytes. (B-D) Relationship between the SLCO4A1 expression level and immunomodulators. (E) Correlation between SLCO4A1 expression level and chemokines. (F) Correlation between SLCO4A1 expression level and receptors.
